# Grape Seeds Proanthocyanidins: An Overview of In Vivo Bioactivity in Animal Models

**DOI:** 10.3390/nu11102435

**Published:** 2019-10-12

**Authors:** Celia Rodríguez-Pérez, Belén García-Villanova, Eduardo Guerra-Hernández, Vito Verardo

**Affiliations:** 1 Department of Nutrition and Food Science, University of Granada, Campus of Cartuja, 18071 Granada, Spain; 2 Institute of Nutrition and Food Technology (INYTA) ‘José Mataix’, Biomedical Research Centre, University of Granada, Avenida del Conocimiento s/n, E-18071 Granada, Spain

**Keywords:** grape seed by-products, proanthocyanidins, metabolic syndrome, chronic diseases, in vivo animal studies

## Abstract

Over the last decade, proanthocyanidins (PACs) are attracting attention not only from the food industry but also from public health organizations due to their health benefits. It is well-known that grapes are a good source of PACs and for that reason, the industry is also focused on grape by-products identification and bioactivity evaluation. Grape seeds extract (GSPE) is a rich source of PACs, mainly composed of monomeric catechin and epicatechin, gallic acid and polymeric and oligomeric proanthocyanidins. Thus, this review encompasses the state-of-art structure and the most recent evidence about the impact of GSPE on chronic diseases, with a focus on oxidative stress, inflammation and metabolic syndrome (MeS)-related disorders such as obesity, diabetes and cardiovascular risk disease in vivo to offer new perspectives in the field that allow further research. Despite the controversial results, is undeniable that PACs from grape seeds are highly antioxidants, thus, the capacity of GSPE to improve oxidative stress might mediate the inflammation process and the progress of MeS-related pathologies. However, further well-design animal studies with standardized dosages and GSPE composition are necessary to shed light into the cause-effect relationship in a more accurate way to later allow a deeper study of the effect of GSPE in humans.

## 1. Introduction

Plant-derived natural products are being actively investigated as health promotors due to their composition in bioactive compounds. Within those bioactive compounds, phenolic compounds represent one of the most interesting group. These biomolecules represent a wide group of secondary plant metabolites playing a crucial role in counteracting various type of stress (ultraviolet irradiation, aggression by pathogens, parasites and plant predators), other than contributing to organoleptic properties of plants and plant-derived food [1,2]. The variety and the high number of compounds from this group is well-known. Proanthocyanidins (PACs), also known as condensed tannins, are spread over the plant kingdom, including fruits, seeds of some plants, flower, nuts or barks. They can be easily found in fruits such as berries or grapes, among others and also in tea, cocoa, chocolate, wine, peanut, almond and avocado or some cereals, among other sources [3,4].

Over the last decade, plant and food-derived PACs are attracting attention not only from the food industry but also from public health organizations due to their ability to prevent chronic diseases [5,6,7]. As examples of the effect of PACs from different matrices is not difficult to find studies regarding juices, syrup and extract cranberry-derived PACs which demonstrated to be effective in the prevention of urinary tract infection in vivo and in vitro [8,9,10]; proanthocyanidins from blueberry-blackberry fermented beverages that showed high antioxidant and anti-inflammatory activity in vitro [11]; or PACs from a persimmon leaf tea that suggested their antihypertensive effect in animal models [12].

More specifically, PACs from grape seeds have shown a broad pharmacological and therapeutic health effects against cardiovascular disease (CVD), diabetes mellitus, obesity or cancer which are related with oxidative stress and inflammatory processes [13,14]. However, the extensive literature available mainly focused on in vitro and in vivo animal models studies, make difficult to have an overview focused on the health effect of grape seed proanthocyanidins. Thus, the aim of this review is to encompass the most recent evidence regarding the in vivo bioactivity of PACs from grape seeds. For that purpose, METLIN and Scopus electronic databases have been employed including literature from the last 10 years related to the effect of grape seeds PACs on oxidative stress, inflammation, obesity, diabetes and cardiovascular risk disease in animal models.

## 2. Composition and Content of Proanthocyanidins in Grape Seeds and Grape Seed Extracts

Winery and grape juice industries produce large amounts of by-products (grape pomace) that are represented by skins, stems and seeds [15]. Grape pomace corresponds to the 20% *w/w* of grape weight and grape seeds constitute until the 52% (*w/w*) of grape pomace [16,17].

Winery and grape juice by-products are a source of phenolic compounds with demonstrated antioxidant and antimicrobial activities [16]; because of that, their revalorization has recently become an area of growing interest [14]. In fact, a considerable amount of available works is focused on the determination of the composition in bioactive compounds of grape by-products such as phenolic compounds. The main grape seeds phenolic compounds include anthocyanins, flavan-3-ols, flavonols, stilbenes, and phenolic acids [18]. Recently, Tang and co-workers [19] noticed that the total flavonoid content in grape seeds is up to ten times higher than grape peel, thus, reinforcing the importance of this grape by-product.

On its behalf, phenolic acids are represented by hydroxybenzoic and hydroxycinnamic derivatives; the most representative of the first class are gallic and protochatechuic acid ranging from 6.5 to 224.0 and from 2.0 to 10.0 µg/g *d.w.*, respectively [17]. The most abundant hydroxycinnamic acids are chlorogenic, caftaric and caffeic acid followed by trans-coutaric acid and their content varied from 0.4 to 68 µg/g *d.w.* [17]. However, ellagic acid derivatives and protocatechuic aldehyde were also determined in enriched grape seed extracts [15].

About flavonols, quercetin and kaempferol derivatives are the most important [15,17,20]; moreover, luteolin and dihydrofisetin derivatives are the most representative flavones [15,16,21] while cyanidin-3-glucoside is the principal anthocyanin determined in grape seed and its content was 0.058–0.840 mg/g *f.w.* [18].

**Figure 1:**
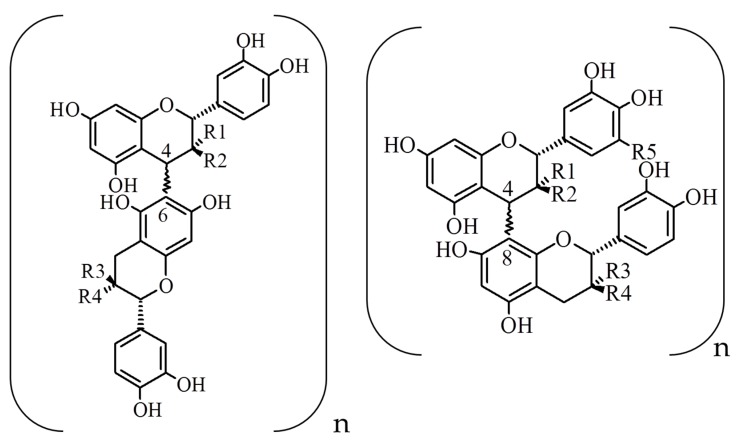
Structural presentation of B-type proanthocyanidin units.

Thanks to the protection against oxidative stress, particular attention was paid to grape seed flavan-3-ols compounds including catechin and epicatechin monomers and their respective oligomers in galloylated and no galloylated form namely proanthocyanidins [22,23,24]. Proanthocyanidins included oligomers and polymers of flavan-3-ol units [25]. According to De Freitas et al. [26], grape seeds contain B-type proanthocyanidins in their galloylated and no-galloylated form. Thus, the flavan-3-ols monomers are singly linked through C4→C6 or C4→C8 bonds [27] (Figure 1).

Several authors studied the grape seed proanthocyanidin composition and their degree of polymerization. Gu et al. [28] reported that the total proanthocyanidins content in grape seeds is 35.3 mg/g of seed *d.w.* and that monomers (catechin and epicatechin) and polymers were the most abundant. Travaglia et al. [29] determined that the mean of proanthocyanidins content in seeds of 37 grape cultivars was 159 mg/g of seed. Escribano-Bailon and co-workers [30] documented that the abundance of PACs in grape seeds are in the following order: (+)-catechin > (-)-epicatechin>(-)-epicatechin-3-O-gallate > epicatechin 3-O-gallate-(4→8)-catechin (B1-3-O-gallate)>epicatechin-(4→8)-epicatechin (dimer B2).

Fuleki and Ricardo da Silva [31] characterized 11 flavan-3-ols in the seeds of 17 grape cultivar showing them (+)-catechin and (−)-epicatechin are the main flavan-3-ols compounds followed by, procyanidin B1, B2, B3, B4, B1-3-O-gallate, B2-3-O-gallate, B2-3‘-O-gallate, and C1.

Bombai and co-workers [32] determined the monomers, oligomers from dimers to dodecamers and polymers. They noticed that monomers are the most concentrated flavan-3-ols in cv. Sangiovese seeds, followed by dimers, trimers and polymers and they found that their concentration was strongly influenced by the ripening. Moreover, Genebra et al. [33] found that the irrigation regime influences the proanthocyanidin biosynthetic pathway more than the genes responsible for their synthesis.

Montero and co-workers [34] also characterized the grape seed proanthocyanidins by 2D-LC-MS identifying from monomers to heptamers and polymers in grape seeds from *cv*. Malvar.

Several approaches were used to collect the proanthocyanidin compounds from grape seeds. Conventionally, organic solvents are used, however, the recoveries are low and unsustainable, thus, advanced green extraction technologies followed by a concentration/purification step are used to produce the extracts for food and health scope [14]. Among others, enzymatic treatment, pulsed electric fields, microwave-assisted extraction, ultrasound-assisted extraction, and supercritical and subcritical fluid extraction are the most studied techniques. However, the industrial application is not still possible for all of them [14,35]. Additionally, it should be mentioned that the concentration/purification step could be carried out by exchange resins purification or by membrane filtration [15,36,37].

Kuhnert et al. [38] analyzed several commercial grape seed extracts by HPLC-FLD and determined a total content of proanthocyanidins ranging from range of 76 to 99%. Polymers (with degree of polymerization > 5) varied from 47 to 81% (*w/w*), monomers and dimers were 7–14% (*w/w*), and galloylated monomers and dimers, trimers and tetramers ranged from 0 to 5.3% (*w/w*). Before establishing a clinical trial, it is very important to evaluate the content of proanthocyanidins in the extracts. In this way, Kuhnert et al. [38] underlined as spectrophotometric assays, which are usually used to determine the total content of proanthocyanidins in grape seed extracts, gives very different results if compared to the HPLC method. Thus, this aspect should be strongly taken into account when standardized grape seed extracts were bought and used to evaluate their in vivo activities.

## 3. Biological Activities of Proanthocyanidins from Grape Seeds

### 3.1. Oxidative Stress

Oxidative stress has been defined as loss of balance between the oxidative and anti-oxidative systems of the cells and tissues producing over production of reactive oxygen species (ROS) [39]. dual-faceted molecules able to influence various physiological cellular processes at modest levels and cause severe oxidative damage to cell components at high levels [40]. It is well known that this imbalance condition plays a crucial role in the pathogenesis of several human diseases such as atherosclerosis, diabetes, hypertension or cancer, among others [12,13,38,41]. In this context, grape seeds proanthocyanidins have demonstrated to be more powerful free radical scavengers than vitamins C, E and β-carotene in vitro and in vivo [13]. A large number of new studies focused on evaluating the role of GSPE against oxidative stress are emerging. Some of the most recent findings are summarized in Table 1. 

Oxidative stress is also related to male infertility through sperm dysfunction caused by an increase of oxygen and oxygen-derived free radicals i.e., ROS [44]. In addition, it has been demonstrated that As and Cd exposition reduces male fertility by peroxidative damage to the plasma membrane [43,47]. In this context, a recent study showed that a dosage of 100/mg/kg BW of GSPE significantly decreased the oxidative stress markers i.e., thiobarbituric acid reactive substance (TBARS), lipid hydroperoxides (LOOH), protein carbonyls (PC), conjugated dienes (CD), and nitric oxide in Cd-induced testes-toxicity rats when administering for 4 weeks [43]. Authors also found a significant decrease of ROS when the extract was employed alone as a treatment and when it was pre-administrated to Cd-treated rats [43,46]. The supplementation with 400 mg/kg/BW of GSPE showed to block As-induced pathological changes and oxidative damage in rats by significantly reducing MDA and 8-OHdG levels and increasing the T-AOC, GSH and SOD activities [47]. In the same line, dosages of 100 and 300 mg/kg BW of GSPE administered to a high-fat diet fed male Sprague Dawley rats significantly decreased malondialdehyde (MDA) levels and increased the glutathione reduced (GSH); glutathione peroxidase (GSH-Px) and superoxide dismutase (SOD) activities of the testes tissue, thus, ameliorating HFD-induced testicular toxicity [44].

Cadmium, together with lead, is one of the most relevant heavy metals which have demonstrated to disrupt the balance of the body’s antioxidant system by reducing the activities of antioxidants enzymes which can induce apoptosis [53]. A clear alteration of several oxidative stress parameters i.e., an increase of MDA levels and a significant decrease of GSH, SOD and γ-GCS activities and the levels of Nrf2 in lung tissue were found in Pb-induced lung toxicity male Wistar rats [48]. However, those parameters were significantly reversed by administering 200 mg/kg BW/day of GSPE for 5 weeks which also decreased the total Pb concentrated in the lung and reduced Pb-induced apoptosis [48]. Moreover, Cd stimulates the formation of metallothioneins and ROS in red blood cells and lymphocytes, thus, inducing erythrocyte apoptosis which can produce anemia [46]. In this regard, Nazima et al. showed that when rats were intoxicated with Cd, the activities of the plasma biomarkers enzymes such as AST, ALT, ACP, LDH and ΥGT increased and ALP activity decreased when comparing to control group. The same trend was found with non-enzymatic antioxidants i.e., TBARS, LH and NO plasma levels that increased in Cd-treated rats in which vitamins C, vitamin E and GSH significantly decreased [46]. Once again, the supplementation GSPE (100 mg/kg BW/day) for 4 weeks significatively prevented the abovementioned impairment, thus, providing protection against Cd-induced oxidative damage in rat erythrocytes [46].

Chronic fluoride intoxication could cause toxicity to the liver [42]. In this regards, earlier research demonstrated that iron overload is implicated in fluoride-induced oxidative damage in vitro attenuated by GSPE which protected the cells against oxidative stress [42]. A more recent study showed that 400 mg/kg BW/day of GSPE significantly decreased levels of ALT, AST, MDA and iron content in liver tissue and increased the GSH-Px, SOD, T-AOC levels in fluoride-induced iron overload Kunming male mice [42]. Despite the mechanism involved in the GSPE liver protection related to fluoride-mediated hepatic oxidative damage is not being fully elucidated, proanthocyanidins from grape seeds have iron-chelating effects that can prevent the production of free radicals normally caused by iron overload [42].

Apart from the protection against oxidative stress induced by metals, medium doses of GSPE (50 and 100 mg/kg BW/day) have also demonstrated anti-fatigue effects in exhaustive exercise-induced fatigue mice by increasing T-AOC, SOD, CAT activities and preventing MDA levels in plasma and skeletal muscle [45]. However, despite at higher doses of 375 mg/kg BW/day, GSPE increased the activity of GSH-Px, no significant changes in MDA liver tissue and plasma ACE were found in spontaneously hypertensive rats [49]. Even so, author highlighted the protective role of GSPE against oxidative stress due to the fast increase of hepatic GSH in treated rats after 6 h post administration [49].

Obesity and related complications such as hypercholesterolemia are linked with higher susceptibility to oxidative stress due to a depletion of antioxidant components such as SOD, GPx, CAT and some vitamins i.e., vitamin A, E, C and β-carotene [50]. Low doses of GSPE (35 mg/kg BW/day) administered for 10 weeks, have also showed an improvement of the hepatic oxidative status in obese Zucker rats through the increment of GSH/GSSG ratio and ORAC and a decrease of GSSG content [52]. Thiruchenduran et al. found a significant increase in SOD, CAT, GSH, ascorbate and α -tocopherol in cardiac tissue from hypercholesterolemic induced-male Wistar rats even with a lower purity extract (37% of PACs) compared to the extracts employed in the other studies [50]. Therefore, PACs from grape seeds decreased lipid peroxidation (LPO) by the chelation of the ROS and increasing the antioxidant defense system [50].

The current literature agrees about the potential protective effect against oxidative stress of GSPE when administered between 35 and 400 mg/kg BW/day in vivo in animal studies. The most recognized mechanism of action resides in the inhibition of lipid peroxidation, avoiding the ROS production, and, therefore, saving cell membranes to apoptosis.

### 3.2. Inflammation

It is not new that flavonoids play an important role in the immune system and, thus, in the inflammatory processes like those related with metabolic syndrome (MeS) such as diabetes, obesity and cardiovascular diseases which are closely associated with inflammatory process [54]. More specifically, PACs from grape seeds were early highlighted to possess anti-inflammatory effects in animal models mainly by inhibiting the formation of pro-inflammatory cytokines [55]. Moreover, it is well-known that a decrease in oxidative stress reduces the inflammatory response but also recent studies have shown that PACs can reduce inflammation in a more direct way as reviewed by Weseler et al. [56]. Table 2 encompasses the latest in vivo studies related to the effect of GSPE on inflammation. It can be seen that doses from 1 to 500 mg/kg BW/day employing extracts from 75 to 95% purity have been tested with promising results.

Several adipokines such as leptin, interleukin 6 (IL-6), tumor necrosis factor alpha (TNF-α), adipsin, resistin and angiotensinogen/PAI-1, and adiponectin, among others are recognized as mediators of inflammation [57]. In inflammation, leptin acts directly on macrophages increasing proinflammatory cytokine production which can be reduced by GSPE [58]. In this context, Terra et al. found that a dose of 345 mg/kg BW/day of GSPE containing mainly oligomeric PACs (31.7%) significantly decreased plasma C-reactive protein (CRP) and increased adiponectin plasma concentrations in male Zucker rats fed with high-fat diet for 19 weeks. Additionally, they found that PACs from grape seed extract modulated the IL-6, TNF-α and adiponectin gene expression in adipose tissue, thus, reducing the diet-induced low-grade inflammation [59]. Interestingly, lower doses of GSPE (20 mg GSPE/animal/day) for a short period of time (10 days) also reduced cytokine expression in liver, muscle and mesenteric adipose tissue [60]. Low doses (20 mg/kg BW GSPE/day) have been administered as part of a preventive treatment in HFD-fed female Wistar rats allowing a significant decrease of CRP, TNF-α, and IL-6 and increasing the anti-inflammatory cytokine adiponectin expression in adipose tissue [58]. A chronical treatment (7 weeks) consisted of 300 mg/kg/BW day of 95% pure GSPE decreased plasma levels of TNF-α, IL-6 and monocyte chemotactic protein 1 (MCP-1) which facilitates the accumulation of macrophages [61]. Nevertheless, GSPE has proved to have anti-inflammatory effects not only at adipose tissue level but also in the intestine, skeletal muscle and lung tissue. At dosages of 100 and 500 mg/kg BW/day, GSPE ameliorated intestinal health in diet-induced obese rats by reducing the proinflammatory cytokine TNF-α secretions to basal levels, reducing transepithelial electrical resistance in small and large intestine and reverting plasma bacterial endotoxins such as lipopolysaccharides to basal levels [62]. Additionally, a down regulation of inflammatory factors such as myeloperoxidase, IL-1β, IL-6 and TNF-α in lung tissue of pulmonary arterial hypertension-induced rats treated with 10 mL/kg BW/day of intraperitoneal 99.5% purity-grape seed proanthocyanidins have been observed [63]. A significant reduction of the expression of TNF-α in the lung was also described when 200 mg/kg BW/day were administered to Pb-induced lung toxicity male Wistar rats [48]. A recent study carried out in mouse model of exhaustive exercise-induced fatigue that treated the animals with GSPE concentrations from 1 to 100 mg/kg BW/day, revealed that medium doses i.e., 50 and 100 mg/kg BW/day reduced TNF-α and IL-1β activities in plasma and in skeletal muscle, thus, improving exhaustive exercise [45].

Even more studies are necessaries, the aforementioned results reinforce the potential of GSPE as anti-inflammatory natural extract capable to downregulate pro-inflammatory cytokines and to increase the expression of the anti-inflammatory ones even at low dosages and using GSP extracts with different composition.

### 3.3. Metabolic Syndrome-Related Disorders

Metabolic syndrome was defined by the World Health Organization (WHO) as a condition that includes abdominal obesity, insulin resistance, hypertension, and hyperlipidemia [64]. The prevalence of MeS is increasing and its prevention has to be multifactorial starting with the change of dietary habits. Since this is a long-term arduous task, several strategies that include the use of pharmaceutical o dietary agents are being tested. In this regard, PAC-rich products e.g., GSPE, berries or *Hibiscus sabdariffa* extract have proved to have anti-obesity effects and/or to improve fatty acid oxidation and insulin responses [65,66]. Concretely, the latest literature focused on the effect of GSPE on metabolic syndrome-related disorders i.e., obesity, diabetes mellitus and cardiovascular risk disease is summarized in Table 3.

#### 3.3.1. Obesity

Obesity increases adipose burden but also alters adipose biology [64]. Proanthocyanidin rich extracts have proved to be involved in obesity modulation through the suppression of food intake and the increase of energy expenditure [63]. In this regard, a recent research concluded that GSPE is capable to modulate the enteroendocrine system in cafeteria-diet-fed rats in which also reduced the BW and food intake at 500 mg/GSPE kg BW per day chronically (17 weeks) [67]. Serrano et al. found that a minimum dose of 350 mg/kg BW was necessary to observe food intake reduction of rats fed with standard chow after an acute oral GSPE treatment [68]. Contrarily, the authors reported that higher doses of GSPE (500 to 1000 mg/kg BW/day) were necessaries for getting an effective effect under a chronic treatment. They found that 79% of food intake could be explained by the hypothalamic glucagon-like peptide 1 expression [68] which has demonstrated to be one of the most promising biological systems related to obesity prevention or treatment [69].

Doses of 345 mg/kg GSPE BW did not influence BW nor adiposity index in HFD-fed Zucker rats [59]. The employed extract was mainly constituted by oligomeric procyanidins (31.7%) followed by monomeric (21.3%), dimeric (17.4%), trimeric (16.3%), tetrameric (13.3%). In agreement, a recent research did not show any change in adiposity at 500 mg/kg BW but they found a significantly decrease of BW in diet-obese Wistar rats using the extract with the same composition [62]. Epidermal fat mass was also reduced in specific-pathogen free male C57BL/6 mice fed with HFD with a >95% purity GSPE extract for 7 weeks without having any effect in BW [61]. Lower doses (300 mg/kg BW) of a GSPE mainly consisted of PAC dimers (56%) also allowed a significant decrease in relative weight of white adipose tissue (WAT) in HFD-fed Sprague Dawley rats [44].

Nevertheless, the mechanism underlying the anti-obesity effect of grape seed PACs has not been fully-elucidated. Margalef et al. concluded that the tissue-detected compounds after the administration of GSPE come from an acute GSPE administration and not from a long-term accumulation [70]. In this regard, the administration of 500 mg/kg BW/day intermittently allowed a significant reduction of diet-induced obese Wistar rats BW [71]. Contrarily, the administration of lower doses of the studied extract i.e., 100 mg/kg BW/day for longer periods of time (30 days) significantly decreased the BW in hypercholesterolemic induced-male Wistar rats [50]. In a more recent study, a significant decrease in food intake accompanied by an increase of energy expenditure in subcutaneous adipose tissue was found in aged male Wistar rats after a sub-chronic intake of 500 mg/kg BW/day for 8 days [71]. Since most of the GSPE have similar but not identical composition, the only factor that seems to be more related to BW decrement could be the employed dose. According to the latest research, the lowest doses employed i.e., 10 or 20 mg/animal/day and 25 mg/kg BW did not show any effect in BW reduction in diet-induced obese Wistar rats [60,72,73,74]. Together with the lack of BW decrement at lower dosages, no significant changes in adiposity index were found in the abovementioned studies. Contrarily, a significant reduction of the adiposity index in hamsters fed with an HFD in which similar dosages (25 mg/kg BW/day GSPE) were employed [75]. Interestingly, Montagut et al. showed a significant reduction on the total amount of visceral adipose tissue using the same dose (25 mg/kg BW/day of GSPE) [72]. It has been shown that the dysregulation of signaling pathway in adipose tissue are associated with insulin resistance that can contribute to the development of obesity-related metabolic disorders [76]. In this regard, Montagut et al. showed GSPE modulated representative markers of mature adipocyte such as Glut4, Srebp1c, Pparg2 after 10 days, presenting a strong down-regulation of those genes after 30 days in induced-obese rats that was stronger at doses of 50 mg GSPE/kg BW/day [72]. On its behalf, Pascual-Serrano exhibited a reversion of adipocyte hypertrophy by a decrease in adipocyte size in visceral WAT [59]. In agreement, a significant reduction of the weight of the WAT depots in hamster fed with a high-fat diet was reported by Caimari et al. [75].

Grape seed proanthocyanidins extract has also proved to be effective anti-obesity effect at low doses when employed as a preventive treatment. Thus, 30 mg/kg BW/day allowed a significant decrease of BW gain when administered for 15 weeks to HFD fed female Wistar rats [60]. Higher concentrations (100 and 300 mg/kg BW) also demonstrated having a protective effect in body weigh increase in HFD-fed male Sprague Dawley rats [44]. There are still discrepancies about the best employed GSPE-based treatment against obesity but some authors suggest that the effectiveness of GSPE supplementation would depend on the initial amount of adipose depots being less effective if the initial adipose tissue is too high [77].

Despite the existing discrepancies regarding the effects of GSPE on fat and body weight, most of the studies have shown an amelioration of the obesity status in vivo when proanthocyanidins from grape seeds were administered. However, the dosage needs to be standardized. In fact, Serrano et al. reinforced the idea that upper effective limit also needs to be also established since they found undesirable effects when GSPE was administered at 1 g/kg BW [68].

#### 3.3.2. Diabetes

As abovementioned, diabetes is closely linked to obesity. In fact, excess accumulation of visceral fat is a high risk for insulin sensitivity. Thus, the prevention of obesity would be directly related to a lower risk of diabetes [64]. Moreover, impaired homeostasis in diabetes mellitus is related to higher production of ROS, consequently, depleting the oxidative stress status. In fact, the long-term oxidative stress caused by hyperglycemia can induce bladder dysfunction that can be prevented administering GSPE (250 mg/kg BW day for 8 weeks) mainly made up 56% dimeric proanthocyanidins, 12% trimeric proanthocyanidins, 6.6% tetrameric proanthocyanidins [78].

Commercial extracts of proanthocyanidins from grape seeds made up monomeric (21.3%), dimeric (17.4%), trimeric (16.3%), tetrameric (13.3%) and oligomeric procyanidins (31.7%) and phenolic acids (4.7%) has been the most employed extract in animal experimental models at doses from 25 to 345 mg/kg BW/day GSPE (Table 3). Low doses of grape seed extract (25 mg/kg BW/day) improved the insulin resistance indexes HOMA-IR, QUICKI and R-QUICKI and the plasma glucose and insulin levels in diet-induced obese male Wistar rats after 3 weeks of treatment [73]. In agreement, the same dose, significantly decreased fasting plasma insulin levels after 10 and 30 days [72]. Higher doses (375 mg/kg BW) also improved significantly the glucose levels in HFD-fed male Zucker rats reducing the glucose levels by 14% [59]. Additionally, the treatment with a different extract composition i.e., 96.64% purity GSPE increased in a significant way the normal insulin content and also the score of beta-cell function and the abnormal oral glucose tolerance were partially reversed [76]. The beta-cell function is involved in folding, export and processing of newly synthesized insulin. The proposed mechanism for which GSPE could avoid beta-cells dysfunction and death in T2DM might be through the decrease of oxidative stress of the endoplasmic reticulum [79]. Those effects could be likely due to the modification of anti-apoptotic markers in the pancreas in animals’ models under altered conditions [80]. Contrarily to the previous reported data, Pascual-Serrano et al. did not find any significant change in glucose, insulin or HOMA-IR plasma levels in diet-induced obese Wistar rats that were treated with different dosages up to 200 mg/kg BW of GSPE for 12 weeks [74].

Other authors, however, showed that the administration of 89% PACs-GSPE (100 mg/kg BW) increased the insulin sensitivity measured by HOMA and QUICKI indices and decreased glucose levels compared to HFD-feed rats but when they compared between treatments, they found that the combination of GSPE and metformin had a better response than individual treatments [81]. The same authors hypothesized that improvement in insulin signaling could be attributed to the reduction of cytokines such as TNF-α and IL-6 in high fat-fructose diet fed Wistar rats when GSPE was administered for 45 days [81].

Notwithstanding most of the experimental studies showed an improvement of glucose homeostasis in animals, there is still no consensus on the chronic effects of proanthocyanidins on the maintenance of whole-body glucose homeostasis as previously affirmed by Montagut et al. [72].

#### 3.3.3. Cardiovascular Risk Disease

According to the WHO, more than 17 million people died from CVDs in 2016 which represent around 31% of all global death (WHO, 2017). Accordingly, hypertension is one of the most important risk factors for CVD being behavioral habits such as diet, sedentarism, and tobacco and alcohol intake implicated in hypertension development. Once again, the diet is one of the essential pillars for the prevention or amelioration of CVD. In this regard, the role of the GSPE has also been studied with very promising results. Quiñones et al. [49] tested three different concentrations of a GSPE i.e., 250, 375 and 500 mg/kg BW in male, spontaneously hypertensive rats. They found a significant decrease in systolic (SBP) and diastolic (DBP) blood pressure which was dose-dependent up to 375 mg/kg BW GSPE in the case of SBP. Interestingly, the effect of the administration of 375 mg/kg BW GSPE in reducing the SBP was similar than the administration of captopril 50 mg/kg BW. Contrarily, no significant differences were found in normotensive Wistar–Kyoto rats supplemented with 375 mg/kg/BW GSPE which suggest that GSPE could be used as treatment but could not have a preventive action against hypertension [49]. Contrarily, Huang et al. showed protective effects against increase of blood pressure in hypertension-induced rats [51]. Additionally, SBP was significantly reduced in rats treated with GSPE as compared to DOCA-salt hypertensive rats from week 3 at doses of 150 mg/kg BW/day and from week 1 at higher doses (240, 384 mg/kg BW/day) together with an increase in aortic rings vasodilatation induced by acetylcholine and a suppression of endothelin-1 plasma and cardiac tissue levels [51]. Remarkably, those effects were comparable with the intake of 5 mg/kg of amlodipine, a specific drug employed for treating hypertension. However, even showing positive effects, the lack of information about the extract composition, make difficult to compare with other studies in order to determine the best composition of GSPE to treat CVDs.

It is known that oxidative stress also plays an important role in the CVD etiology in spite that the direct mechanism of action is still being studied [82]. Some studies show that doses from 150 to 384 mg/kg BW/day GSPE increase SOD activities, inhibit the increase of serum and cardiac MDA and also inhibit the p-JNK1/2 and p-p38MAPK expressions, mainly implicated in cardiomyocyte hypertrophy [51,83].

On the other hand, hyperlipidemia, related to refer to elevated cholesterol, elevated TAGs or both, is also pointed out as one of the CVD risks factor which is also closely related to the diet [84]. In this context, Thiruchenduran, et al. [50] showed that the administration of 100 mg/kg BW of GSPE, with a purity of 37%, significantly reversed the serum cholesterol, LDL, free fatty acids and TAGs levels while increased serum phospholipids and HDL concentration in experimentally induced hypercholesterolemia Wistar rats. Additionally, the same authors found an amelioration in the activity of diagnostic cardiac serum markers enzymes such as glutamate oxaloacetate transaminase (SGOT), lactate dehydrogenase (LDH) and creatine kinase (CK) [50]. Lower doses (25 mg GSPE/kg BW) of GSPE made up flavan-3-ols (21.3%), dimers (17.4%), trimers (16.3%), tetramers (13.3%) and oligomers (31.7%) of PACs allowed a significant decreased of TC by around 20% but not in TAG or LDL in diet-induced obese Wistar rats [73]. Surprisingly, the decreased TC plasma levels were observed when GSPE was administered for 3 weeks [73] but not when the supplementation was for longer (12 weeks) [74]. This suggests that the time of the treatment could influence in a great manner the effect of the treatment. Contrarily, higher doses (from 100 to 345 mg/kg BW/day) of the same GSP extract did not show any significant change in TC, TAGs or HDL plasma levels when administered chronically, i.e., from 3 to 12 weeks [74] nor intermittently at even higher doses (500 mg/kg BW/day) [77].

Due to the controversial results, further research is necessaries to stablish a cause-effect relationship between the administration of GSPE and the prevention or amelioration of CVD risks. It is noteworthy to mention that, even most studies employed similar doses of GSPE, the difference between results might be due to the different composition of the grape seed extracts in PACs. In fact, despite most of the studies administered commercial extracts, some others employed in-house extracts without an in deep characterization of the PAC content [50], thus, making difficult the comparison between them.

## 4. Conclusions

The interest of natural products to treat and/or prevent chronic diseases is increasing. In this regard, much evidence, both, in vitro and in animal models has been accumulated regarding the relationship between the antioxidant effect of natural products and the development of chronic diseases. In fact, a large number of research studies have demonstrated a broad spectrum of pharmacological and therapeutic benefits of GSPE. Indeed, is undeniable that PACs from grape seeds are highly antioxidants. Accordingly, since oxidative stress, inflammation process and MeS-related pathologies are connected somehow, GSPE bioactivity could be mainly mediated by its antioxidant activity. However, contradictory conclusions resulted from the use of different animal species, small sample size, different GSPE doses and duration of the studies make complicated to stablish the real bioactive effect of GSPE on health. In this regard, the standardization of the dosage and the composition of the extracts is a necessity to shed light into the cause-effect relationship between the intake of PACs from grape seeds and their health effects in a more accurate way. It should not be forgotten that the synergistic effect of GSPE and other already employed drugs should be noteworthy and further studied.

Despite GSPE have proven to be a promising natural extract with multiple health benefits in in vivo animal studies, further well-design clinical trials are necessaries to confirm the beneficial effects of GSPE on oxidative stress, inflammation and MeS-related pathologies such as obesity, diabetes and CVD in humans.

## Figures and Tables

**Table 1:** Effect of proanthocyanidins from grape seeds on oxidative stress in animal models.

GSPE Extract Composition	GSPE Purity *	Dose	Study Design	Major Outcomes	Reference
Oxidative stress					
NE	99%	400 mg/kg BW/day	Fluoride-induced iron overload Kunming male mice; GSPE (5 weeks)	↓ ALT, AST, MDA, iron content; ↑ GSH-Px, SOD, T-AOC	[42]
54% dimeric, 13% trimeric procyanidins, and 7% tetrameric proanthocyanidins	NE	100 mg/kg BW	Cd intoxicated-adult male albino Wistar rats; GSPE (4 weeks)	↓ ROS, TBARS, LOOH, PC, CD, and NO, ↑ GSH, TSH, Vitamin C and E	[43]
Dimer (56%), trimer (12%), tetramer (6.6%), monomers and other high-molecular mass oligomers (20.4%)	>95%	100 and 300 mg/kg BW	HFD fed male Sprague Dawley rats; GSPE (13 weeks)	↓ MDA levels and ↑ GSH, GSH-Px and SOD activities of the testes tissue	[44]
NE	≥95%	1, 50 and 100 mg/kg BW/day	Male ICR mice; GSPE (28 days)	↑ T-AOC, SOD, CAT and ↓ MDA activities in plasma and in skeletal muscle (50 and 100 mg/kg BW day of GSPE)	[45]
54% dimeric, 13% trimeric and 7% tetrameric proanthocyanidins	NE	100 mg/kg BW/day	Cd-intoxicated male albino Wistar rats; GSPE (4 weeks)	↓ AST, ALT, ACP, LDH and ΥGT and ↑ ALP; ↓ TBARS, LH, NO plasma levels and ↑ vitamins C and E and GSH plasma concentration	[46]
NE	>95%	High dose 400 mg/kg BW/day, 100, 200, 400 mg/kg BW/day	As-induced Oxidative Reproductive Toxicity male Kunming mice; GSPE (5 weeks)	GSPE (400 mg/kg/BW) ↓ MDA and 8-OHdG levels and ↑ T-AOC, GSH and SOD activities	[47]
56% dimeric, 12% trimeric procyanidins, and 6.6% tetrameric PACs	NE	200 mg/kg BW/day	Pb-induced lung toxicity male Wistar rats, GSPE (5 weeks)	↓ MDA levels and total Pb accumulation in the lung. ↑ GSH, SOD, γ-GCS activities and Nrf2 levels in lung tissue	[48]
Flavan-3-ols (21.3%), dimers (17.4%), trimers (16.3%), tetramers (13.3%) and oligomers (5–13 units; 31.7%) of PACs	>75%	375 mg/kg BW/day	Male, spontaneously hypertensive rats, GSPE (2 days)	No changes in MDA liver tissue and plasma ACE, ↑ GSH	[49]
NE	37%	100 mg/kg BW/day	Hypercholesterolemic induced-male Wistar rats; GSPE (30 days)	↑ SOD, CAT, GSH, ascorbate and α -tocopherol in cardiac tissue	[50]
NE	NE	150, 240, 384 mg/kg BW/day	DOCA hypertension-induced SD rats; GSPE (4 weeks)	↑ SOD activities, inhibition of the increase of serum and cardiac tissue MDA, inhibition of p-JNK1/2 and p-p38MAPK	[51]
Catechin (58 mol/g), epicatechin (52 mol/g), epigallocatechin (5.50 mol/g), epicatechin gallate (89 mol/g), epigallocatechin gallate (1.40 mol/g), dimeric procyanidins (250 mol/g), trimeric procyanidins (15.68 mol/g), tetrameric procyanidins (8.8 mol/g), pentameric procyanidins (0.73 mol/g), and hexameric procyanidins (0.38mol/g)	>75%	35 mg/kg BW/day	Obese Zucker rats; GSPE (10 weeks)	↑ GSH/GSSG ratio and ORAC, ↓ GSSG content	[52]

* NE, non-specified; ACE, plasma angiotensin-converting enzyme activity; ALP, alkaline phosphatase; AST, aspartate aminotransferase; BW, body weight; CAT, catalase; CD, Conjugated dienes; γ-GCS, γ-glutamyl cysteine synthetase; GSH, glutathione reduced; GSH-Px, glutathione peroxidase; GSPE, grape seed proanthocyanidins extract; GSSG, Glutathione disulphide; γGCS, γ-glutamate-cysteine ligase; γGT, gamma-glutamyl transferase; HFD, high-fat diet; LDH, lactate dehydrogenase; LOOH, lipid hydroperoxides; MDA, malondialdehyde; NO, nitric oxide; 8-OHdG, 8-hydroxy-2′-deoxy guanosine; ORAC, oxygen radical absorbance capacity; p38MAPK, p38 mitogen-activated protein kinases; PACs, proanthocyanidins; PC, protein carbonyls; p-JNK1/2, JNK1/2 phosphorylation; SOD, superoxide dismutase; T-AOC, total antioxidant capacity; TBARS, thiobarbituric acid reactive substances.

**Table 2:** Effect of proanthocyanidins from grape seeds on inflammation in animal models.

GSPE Extract Composition	GSPE Purity*	Dose	Study Design	Major Outcomes	Reference
Inflammation					
NE	>95%	300 mg/kg BW/day	Specific-pathogen free male C57BL/6 mice fed with HFD; GSPE (7 weeks)	↓ Plasma levels of TNF-α, IL-6 and MCP-1	[61]
Flavan-3-ols (21.3%), dimers (17.4%), trimers (16.3%), tetramers (13.3%) and oligomers (5–13 units; 31.7%) of PACs	>75%	345 mg/kg BW/day	HFD fed male Zucker rats; GSPE (19 weeks)	↓ CRP, ↑ adiponectin plasma levels, no differences in IL-6 plasma levels	[59]
Flavan-3-ols (21.3%), dimers (17.4%), trimers (16.3%), tetramers (13.3%) and oligomers (5–13 units; 31.7%) of PACs	>75%	30 mg/kg BW/day	HFD fed female Wistar rats; GSPE (15 weeks) - Preventive treatment	↓ CRP and TNF-α plasma and adipose tissue levels, ↓ IL-6, Emr1 and ↑ adiponectin in adipose tissue	[60]
Flavan-3-ols (21.3%), dimers (17.4%), trimers (16.3%), tetramers (13.3%) and oligomers (5–13 units; 31.7%) of PACs	>75%	10 or 20 mg/animal/day	Diet-induced obese female Wistar rats; GSPE (10 days or 30 days)	↓ CRP and TNF-α plasma levels after 10 days (20 mg/animal/day)	[60]
Flavan-3-ols (21.3%), dimers (17.4%), trimers (16.3%), tetramers (13.3%) and oligomers (5–13 units; 31.7%) of PACs	>75%	100 and 500 mg/kg BW/day	Diet-induced obese female Wistar rats; GSPE (2 weeks)	↓ TNF-α secretions, ↓ transepithelial electrical resistance in small and large intestine, ↓ plasma LPS to basal levels	[62]
56% dimeric, 12% trimeric procyanidins, and 6.6% tetrameric PACs	NE	200 mg/kg BW/day	Pb-induced lung toxicity male Wistar rats, GSPE (5 weeks)	↓ inflammatory cells in the lung tissue, ↓ TNF-α in lung tissue	[48]
NE	≥95%	1, 50 and 100 mg/kg BW/day	Male ICR mice; GSPE (28 days)	↓ TNF-α and IL-1β activities in plasma and in skeletal muscle (50 and 100 mg/kg BW day of GSPE)	[45]
NE	99.5 g GSP/mL	10 mL/kg BW/day	Monocrotaline-induced PAH male Sprague–Dawley rats; GSPE (3 weeks)	Down regulation of myeloperoxidase, IL-1β, IL-6 and TNF-α in lung tissue	[63]

* NE, non-specified; BW, body weight; CRP, C-reactive protein; Emr1, mucin-like hormone receptor 1; GSP, grape seed proanthocyanidins, GSPE, grape seed proanthocyanidins extract; IL, interleukin; LPS, lipopolysaccharides; MCP-1, monocyte chemoattractant protein 1; PACs, proanthocyanidins; PAH, pulmonary arterial hypertension; TNF-α, tumor necrosis factor alpha.

**Table 3:** Effect of proanthocyanidins from grape seeds on metabolic syndrome-related diseases in animal models.

GSPE Extract Composition	GSPE Purity *	Dose	Study Design	Major Outcomes	Reference
**Obesity**					
Flavan-3-ols (21.3%), dimers (17.4%), trimers (16.3%), tetramers (13.3%) and oligomers (5–13 units; 31.7%) of PACs	>75%	500 mg/kg BW/day	Aged male Wistar rats; GSPE (8 days)	↓ Food intake, ↑ energy expenditure, ↓ BW	[71]
Flavan-3-ols (21.3%), dimers (17.4%), trimers (16.3%), tetramers (13.3%) and oligomers (5–13 units; 31.7%) of PACs	>75%	Acute treatment 1000 mg/kg; Chronic treatment: 500 and 1000 mg/kg BW/day	Female Wistar rats; GSPE (8 days)	Acute treatment: ↑ GLP-1 plasma levels, CART mRNA expression; chronic treatment: no differences in leptin levels; no clear effects on the hypothalamic mRNA levels	[68]
Flavan-3-ols (21.3%), dimers (17.4%), trimers (16.3%), tetramers (13.3%) and oligomers (5–13 units; 31.7%) of PACs	>75%	100 and 500 mg/kg BW/day	Diet-induced obese female Wistar rats; GSPE (2 weeks)	No changes in adiposity, ↓ BW gain at 500 mg/kg/BW	[62]
Catechin (121 ± 3 mg/g), epicatechin (93 ± 4 mg/g), PAC dimer B1 (89 ± 3 mg/g), PAC dimer B3 (46 ± 2 mg/g)	NE	25 mg/kg BW/day	Diet-induced obese male Wistar rats; GSPE (3 weeks)	↓ adipocyte size, no reduction of BW gain, no reversion of adiposity in WAT	[73]
Phenolic acids (1.63%), as well as monomeric (20.9%), dimeric (20.7%), trimeric (17.3%) and oligomeric (39.41%) procyanidins.	NE	25 mg/kg BW/day	Male Golden Syrian hamsters fed with HFD; GSPE (15 days)	↓ Adiposity index, the weight of the WAT depots and the BW gain	[75]
Dimer (56%), trimer (12%), tetramer (6.6%), monomers and other high-molecular mass oligomers (20.4%)	>95%	300 mg/kg BW	HFD fed male Sprague Dawley rats; GSPE (13 weeks)	↓ Relative weight of WAT	[44]
NE	>95%	300 mg/kg BW/day	Specific-pathogen free male C57BL/6 mice fed with an HFD; GSPE (7 weeks)	↓ Epidydimal fat mass, no changes in BW	[61]
NE	37%	100 mg/kg BW/day	Hypercholesterolemic induced-male Wistar rats; GSPE (30 days)	↓ BW	[50]
Flavan-3-ols (21.3%), dimers (17.4%), trimers (16.3%), tetramers (13.3%) and oligomers (5–13 units; 31.7%) of PACs	>75%	25 mg/kg BW/day	Diet-induced obese female Wistar rats; GSPE (10 days and 30 days)	↓ the total amount of visceral adipose tissue, no reduction of BW gain, no changes in plasma leptin levels after 30 days intervention	[72]
Flavan-3-ols (21.3%), dimers (17.4%), trimers (16.3%), tetramers (13.3%) and oligomers (5–13 units; 31.7%) of PACs	>75%	345 mg/kg BW/day	HFD fed Male Zucker rats; GSPE (19 weeks)	No changes in adiposity index or in BW	[59]
Monomeric (21.3%), dimeric (17.4%), trimeric (16.3%), tetrameric (13.3%) and oligomeric (5–13 units) (31.7%) procyanidins and phenolic acids (4.7 %)	>75%	30 mg/kg BW/day	HFD fed female Wistar rats; GSPE (15 weeks) - Preventive treatment	↓ BW gain, no changes in adiposity or the weight of fat depots	[60]
Flavan-3-ols (21.3%), dimers (17.4%), trimers (16.3%), tetramers (13.3%) and oligomers (5–13 units; 31.7%) of PACs	>75%	10 or 20 mg/animal/day	Diet-induced obese female Wistar rats; GSPE (10 days or 30 days)	No significant ↓in adiposity index or in BW	[60]
Flavan-3-ols (21.3%), dimers (17.4%), trimers (16.3%), tetramers (13.3%) and oligomers (5–13 units; 31.7%) of PACs	>75%	25 mg/kg BW	Diet-induced obese male Wistar rats; GSPE (12 weeks)	No significant reduction in weight gain or reverse or adiposity, ↓ adipocyte size	[73]
Flavan-3-ols (21.3%), dimers (17.4%), trimers (16.3%), tetramers (13.3%) and oligomers (5–13 units; 31.7%) of PACs	>75%	25, 100 and 200 mg/kg BW	Diet-induced obese male Wistar rats; GSPE (3 weeks)	Not improvement of adiposity index, prevention in the increase of the area and volume of the WAT, no change in leptin plasma levels, BW and upregulation PPARΥ (200 mg/kg BW)	[74]
Monomers of flavan-3-ols (21.3%), dimers (17.4%), trimers (16.3%), tetramers (13.3%) and oligomers (5–13 units; 31.7%) of PACs	>75%	500 mg/kg BW/day	Diet-induced obese female Wistar rats; GSPE intermittently	↓ BW, total WAT, BAT, % visceral adiposity and % total adiposity	[77]
**Diabetes**					
6.1% catechin, 6.78% epicatechin, 55.59% dimeric forms, 11.91% trimeric forms, 6.55% tetrameric forms and small amounts of other polymeric forms	96.64%	500 mg/kg BW/day	STZ-induced diabetic male Sprague–Dawley rats with basal diet; GSPE (16 weeks)	The score of beta-cell function and the abnormal oral glucose tolerance partially reversed, ↑ normal insulin content	[76]
Monomeric (21.3%), dimeric (17.4%), trimeric (16.3%), tetrameric (13.3%) and oligomeric (5–13 units; 31.7%)	>75%	25 mg/kg BW/day	Diet-induced obese female Wistar rats; GSPE (10 days and 30 days)	↓ fasting plasma insulin levels after 10 and 30 days	[72]
Monomeric (21.3%), dimeric (17.4%), trimeric (16.3%), tetrameric (13.3%) and oligomeric (5–13 units) (31.7%) procyanidins and phenolic acids (4.7%)	NE	345 mg/kg BW/day	HFD fed Male Zucker rats; GSPE (19 weeks)	↓ glucose levels	[59]
89 % PAC, 6 % monomers, and 5 % other materials	NE	100 mg/kg BW/day	High fat-fructose diet fed male Wistar rats; GSPE (45 days)	↓ glucose and insulin levels, ↑ insulin sensitivity, restoration of the activities of glycolytic enzymes in the liver	[81]
Flavan-3-ols (21.3%), dimers (17.4%), trimers (16.3%), tetramers (13.3%) and oligomers (5–13 units; 31.7%) of PAs	>75%	25, 100 and 200 mg/kg BW	Diet-induced obese male Wistar rats; GSPE (12 weeks)	No significant changes in glucose, insulin or HOMA-IR plasma levels	[74]
Flavan-3-ols (21.3%), dimers (17.4%), trimers (16.3%), tetramers (13.3%) and oligomers (5–13 units; 31.7%) of PAs	>75%	25 mg/kg BW/day	Diet-induced obese male Wistar rats; GSPE (3 weeks)	↓ plasma glucose and insulin levels	[73]
**Cardiovascular risk disease**					
Flavan-3-ols (21.3%), dimers (17.4%), trimers (16.3%), tetramers (13.3%) and oligomers (5–13 units; 31.7%) of procyanidins.	>75%	375 mg/kg BW	Male, spontaneously hypertensive rats; GSPE (2 days)	↓ DBP and SBP	[49]
NE	37%	100 mg/kg BW	Hypercholesterolemic induced-male Wistar rats; GSPE (30 days)	↓ Tissue and serum cholesterol levels, LDL, serum free fatty acids, serum TAGs and ↑ (*p* < 0.01) serum phospholipids and HDL.	[50]
Flavan-3-ols (21.3%), dimers (17.4%), trimers (16.3%), tetramers (13.3%) and oligomers (5–13 units; 31.7%) of PAs	>75%	25 mg/kg BW/day	Diet-induced obese male Wistar rats; GSPE (3 weeks)	↓ TC	[73]
Flavan-3-ols (21.3%), dimers (17.4%), trimers (16.3%), tetramers (13.3%) and oligomers (5–13 units; 31.7%) of PACs	>75%	345 mg/kg BW/day	HFD fed Male Zucker rats; GSPE (19 weeks)	No changes in total plasma cholesterol	[59]
NE	NE	150, 240, 384 mg/kg BW/day	DOCA hypertension-induced SD rats; GSPE (4 weeks)	↓ SBP, reversion of morphological hypertrophy of heart, ↑ in aortic rings vasodilatation	[51]
Flavan-3-ols (21.3%), dimers (17.4%), trimers (16.3%), tetramers (13.3%) and oligomers (5–13 units; 31.7%) of PACs	>75%	25, 100 and 200 mg/kg BW	Diet-induced obese male Wistar rats; GSPE (12 weeks)	No significant changes in plasma TC, TAGs, HDL	[74]
Flavan-3-ols (21.3%), dimers (17.4%), trimers (16.3%), tetramers (13.3%) and oligomers (5–13 units; 31.7%) of PACs	>75%	500 mg/kg BW/day	Diet-induced obese female Wistar rats; GSPE intermittently	No significant changes in plasma TAGs, fatty acids and cholesterol levels	[77]

* NE, non-specified; BAT, brown adipose tissue; BW, body weight; CART, Cocaine- and amphetamine-regulated transcript; DBP, diastolic blood pressure; GLP-1, glucagon-like peptide-1; GSPE, grape seed proanthocyanidins extract; HDL, high-density lipoprotein; HFD, high-fat diet; HOMA-IR, homeostasis assessment model for insulin resistance; LDL, low-density lipoprotein; Nrf2, Nuclear factor erythroid 2-related factor 2; PACs, proanthocyanidins; PPARΥ, Proliferator-activated receptor gamma; SBP, systolic blood pressure; TAGs, triglycerides; TC, total cholesterol; WAT, white adipose tissue.
